# Dental anxiety in patients attending a student dental clinic

**DOI:** 10.1186/s12903-018-0507-5

**Published:** 2018-03-20

**Authors:** Marie L. Caltabiano, Felicity Croker, Lauren Page, Anton Sklavos, Jade Spiteri, Louise Hanrahan, Richard Choi

**Affiliations:** 1 Psychology, College of Healthcare Sciences, James Cook University, McGregor Road, Smithfield, Cairns, Qld 4878 Australia; 2 College of Medicine & Dentistry, James Cook University, Cairns, QLD Australia

**Keywords:** Dental anxiety, Student dentist, University dental clinic, Australia

## Abstract

**Background:**

This study investigated the expectations and experiences of a sample of new patients visiting an Australian regional university Student Dental Clinic with regard to anxiety provoking and alleviating stimuli in the clinical environment. Differences in anxiety levels were examined by age, gender and the type of procedure undergone.

**Methods:**

The number of dental patients who participated in the study was 102 (56 males, 43 females). The study used a pre-treatment/post-treatment design to assess the effect of the dental procedure on anxiety levels of patients. The Modified Dental Anxiety Scale (MDAS) was used to measure anxiety levels in patients at pre-treatment. Questions were also asked about factors which may increase (length of the appointment, invasiveness of procedure) or decrease (perceived student interpersonal skills and clinical ability) dental fear.

**Results:**

Females reported higher total MDAS scores (M = 11.93) compared to males (M = 9.94). Younger patients (M = 12.15) had higher dental anxiety than older patients (M = 9.34). There was a reduction in dental anxiety from pre-treatment (M = 1.92) to post-treatment (M = 1.23) on the single item anxiety measure though most of the treatment being undergone by patients was for less complex procedures.

**Conclusions:**

Patients’ anticipatory experience of anxiety was higher than the anxiety experience after having undergone treatment at the student dental clinic. Student interpersonal skills and clinical ability as perceived by the patient can lessen dental anxiety in patients. Clinical Supervisor-student ratios need to be more equivalent in order to reduce the time length of appointments which currently are associated with increased patient anxiety levels in student dental clinics.

## Background

Dental anxiety can be described as an aversive emotional state of apprehension or worry in anticipation of the feared stimulus of dental treatment [1]. Dental anxiety has been found to play a central role in the avoidance of dental treatment [2]. Researchers have described the cycle of dental avoidance whereby dentally anxious people avoid dental care and hence leave their oral health issues to worsen [3, 4]. Poor oral health results in shame and avoidance of the dentist until the experience of pain or unbearable symptoms drives the patient to seek treatment [4]. This pattern reinforces the fear of dental treatment and feelings of dental anxiety. A recent study confirmed that those who have high levels of dental fear have poor oral health habits (infrequent tooth brushing, tobacco use, unhealthy eating habits) which increase the need for treatment at checkups [5].

A considerable amount of research has focused on dental anxiety in adult clinical, college and community samples from various countries [6–10]. In Australian adults the prevalence of dental anxiety has been cited as approximately 16% [11].While there is some evidence for dental anxiety to decline with age [6, 9], the influence of age on dental anxiety and dental attendance has not been conclusive. One Australian study [3] reported that those in the 18–34 year age bracket had lower levels of dental anxiety than those aged between 35 and 44 years. Findings from *the Australian Research Centre for Population Oral Health* established that the 45–54 age bracket were less likely to report infrequent dental visits than all other age groups [12]. The differences in findings can be attributed to different samples, and different measures of dental anxiety.

Other characteristics of dentally anxious people have been documented. In terms of gender, females have been consistently identified as having greater levels of dental anxiety than males [2]. This gender difference in dental anxiety holds at different age levels [6]. Epidemiological research on middle-aged and elderly women [9] found that both dental anxiety and regular dental attendance declines with increasing age in dentate women. The oral status of dentally anxious women who visited the dentist regularly was better (more preserved teeth, less caries lesions, more restorations) than those with irregular attendance.

There has been some work published regarding whether or not the dental treatment received influences patient anxiety levels. Early research established that painful dental experiences and expectations of trauma were associated with fear of dental procedures [13]. These negative reactions were countered by personal qualities of the dentist [13]. Procedures involving the needle or drill seem to evoke the most fear. Invasive procedures such as subgingival scaling, deep probing, fillings, extractions and root canal therapy are associated with more reported pain, especially in those with high dental anxiety [14]. These patients also had previous painful experiences, expected treatment to be painful and reported lower levels of control during treatment. In a recent study [8] involving a patient group receiving dental hygiene maintenance, those with higher dental anxiety anticipated more pain from procedures involving probing, scaling and vibrating sensations. The literature however indicates that dental fear is lessened prophylactically through regular dental visits [15].

Dental practitioners and dental students will be familiar with dental anxiety and aware of the barriers it poses to treatment outcomes. Implications that arise when treating the anxious patient can include; more appointment cancellations, patients failing to attend altogether, impaired health outcomes and heightened perceptions of pain [1, 16–18]. A recent 2016 study [19] has found support for sequential stages involving behavioural, cognitive and emotional psychological factors in the decision to attend a dental appointment. Negative evaluations of previous dental experiences influence behavioural intentions to visit the dentist via their influence on expectations of an unpleasant or painful dental experience. While dental anxiety determined intentions to visit the dentist, its influence became non-significant when previous dental experience, and future expectations where entered into the predictive model. There is some evidence that prior dental experience is not always remembered accurately. In a study on patients who had undergone tooth extraction, recalled pain one month post-extraction was greater than the pain reported at the time of the procedure, especially in those with higher levels of dental anxiety [20].

Australian undergraduate dentistry students in their clinical years, practice under the supervision of experienced dental professionals. Members of the general public are welcomed into these facilities as patients, with many receiving treatment for free or at a discounted cost. Given that compromised dental health as a result of dental anxiety and infrequent dental visits continues to be an issue, it is important to know the levels of anxiety experienced by patients who choose to visit a student dental clinic.

There have been some studies which have investigated dental anxiety in adult patients attending a dental school clinic. In one study [21] dental anxiety was found to be higher among patients attending a dental school emergency clinic compared to general population samples, with those seeking care infrequently having higher anxiety. Woodmansey [22] found low to medium anxiety in his sample of patients attending a university dental clinic whether assessed using the Corah Dental Anxiety Scale or the one item Dental Anxiety question (DAQ) ‘Are you afraid of going to the dentist?’ Another study [23] reported high dental anxiety in 13.6% of their sample using the MDAS. However, analyses were based on combined data from four clinics one of which was a university dental school, private and public hospital clinics in Ghana, so that it is difficult to know whether there were differences between patients in the different types of clinics. In a Turkish study [24] of patients attending a dental school clinic, 21.6% had both high trait anxiety and dental anxiety, though neither types of anxiety were found to be associated with the number of decayed, missing and filled teeth. An Iranian study [10] on the experiences of patients visiting a Dental School reported 58% to have dental anxiety. Dental anxiety was higher in women, but no differences were found for age or education. Those who visited the dentist regularly and who did not have previous traumatic experiences were less dentally anxious. The study however did not explore the type of treatment received or contextual variables associated with the dental environment.

The aim of this study was to examine the prevalence of dental anxiety before and after treatment in a sample of patients seeking treatment in a student dental clinic. Demographic factors (age, gender) and factors that may increase (length of the appointment, invasiveness of treatment) or decrease (perceived student interpersonal skills and clinical ability of the student dentist) dental fear were also examined.

It was hypothesised that 1) patients at the Student Dental Clinic would experience higher levels of anxiety on the Modified Dental Anxiety Scale (MDAS) compared to normative data on the MDAS [25]; 2) female patients would be more anxious than male patients; 3) younger patients would have more dental anxiety; 4) there would be a reduction in patient anxiety from pre-treatment to post-treatment on the single MDAS item regarding anxiety while in the waiting room and anxiety after the treatment; and 5) complexity of treatment would be associated with higher levels of anxiety.

## Methods

### The student dental clinic

The Australian regional university where the data for the present study was collected commenced its Bachelor of Dental Surgery in 2009, with the opening of its Student Dental Clinic, in 2011. The clinic is open to the public, with the service being free of charge to Australian Healthcare cardholders.

### Study design

This study assessed anxiety levels of new patients visiting the Student Dental Clinic at pre-treatment and post-treatment. Data was collected on each Friday over a two month period. The student dentists treating patients were mostly Level 3 students who practice comprehensive dentistry, and were chosen as they see a higher proportion of new patients. Only Level 4 students are permitted to do molar RCT, crown and bridge work in addition to more complex extractions. Where these more complex procedures were required Level 4 students performed them. The students who administered the questionnaires were Level 4 students. However, in order to prevent any bias the interviewer students were not the student dentists performing the dental procedures on patients. The student dentists were aged between 20 and 21 years and both genders were represented.

### Patient characteristics

To be included in the study, participants were required to be new patients of the Student Dental Clinic, aged 18 years or above. Participants were required to be able to speak English, have the capacity to give consent, be willing to complete the requested surveys and share their thoughts and experiences. There were 102 study participants, 56 of whom identified as male, 43 of whom identified as female and 3 of whom did not disclose their gender. All new patients who were approached to participate and who met the inclusion criteria agreed to do so. Data collection was stopped when the required sample size was reached. The reader is referred to the section on Data Analysis and use of G*Power to estimate respondent sample. The study sample represented 1/5 of all new adult patients over the two month period.

### Measurements

The pre-treatment questionnaire used was the Modified Dental Anxiety Scale (MDAS) [26]; a set of five questions with response format (1 = not anxious, 2 = slightly anxious, 3 = fairly anxious, 4 = very anxious, 5 = extremely anxious) a validated and reliable scale with extensive normative data from different countries [25–27]. The five questions assess anxiety in relation to having treatment tomorrow, sitting in the waiting room, having a tooth drilled, having teeth scaled and polished and having a local anaesthetic injection. Scores are added across all items with the highest possible score being 25. A cut-off score of 19 on the MDAS is indicative of high dental anxiety [25]. The MDAS was specifically selected due its ease of use and the minimal amount of time required for its completion. Participants were also asked to indicate their age, gender and ethnicity.

The post-treatment questionnaire also consisted of five questions, designed specifically for the minimal amount of time required for its completion. The first question asked ‘How anxious are you feeling at the moment?’ and was rated on a 5-point scale from not anxious to extremely anxious reflecting the MDAS response format ie. 1 = not anxious, 2 = slightly anxious 3 = fairly anxious 4 = very anxious 5 = extremely anxious. Questions two and three asked participants which, if any, contextual factors made them more or less anxious during their appointment. These contextual factors were Time length of appointment, Clinical environment, perceived Interpersonal skill of the student, perceived Clinical ability of the student, Presence of the supervisor, Having to participate in the procedure (holding suction), Knowledge of additional appointments, None of the above, and Other (please specify). Respondents were able to select more than one of the factors. Question four asked participants to indicate which procedures, if any, they received during their appointment. Table 4 lists the treatments received by patients. The final question was open-ended and asked for any other comments regarding their experience at the clinic. On both questionnaires a participant code was used to enable pairing of the questionnaires for analysis.

### Procedure

Adult participants were recruited on arrival at the Student Dental Clinic prior to the commencement of their first appointment. An information sheet and consent form were provided to all potential participants. Participants were then able to place their consent forms into a secure locked box if they wished to participate. All consenting participants were then asked to complete a short, self-administered questionnaire before they commenced treatment, which allowed the anxiety levels of patients to be identified prior to their experience at the Student Dental Clinic. Upon being escorted out of the clinic and into the waiting room, participants were greeted by one of the investigators and given a post- appointment questionnaire which consisted of five questions regarding their experience. Investigators were present at the Student Dental Clinic for the completion of all participants’ first appointments to assist with any queries and ensure all surveys were collected and stored in a secured box. Participants were offered the option of completing both of the two surveys in the privacy of an interview room separated from the waiting room.

### Data analysis

The data collected was analysed using the Statistical Package for the Social Sciences (SPSS) Version 22 [28]. T-tests were used to analyse the data for gender differences. A one-way Analysis of Variance (ANOVA) was used to assess age differences in dental anxiety for different age groupings. A Repeated Measures Analysis of Variance was used to examine pre-post treatment differences on dental anxiety. Reliability analysis for the Modified Dental Anxiety Scale (MDAS) was conducted using Cronbach’s Alpha. The probability level of *p* < .05 was selected for statistical significance. Means and standardised deviations were used to compare the current data with existing norms.

A Power analysis was initially undertaken to determine the number of participants which are required to detect an effect of a given size ie. the magnitude of the difference between groups or between persons over time. G*Power software [29] was used to calculate statistical power for ANOVA tests for an effect size of 0.8 and .05 level of statistical significance, indicating a required sample size of 100 participants. If two groups’ means do not differ by 0.2 standard deviations or more the difference is trivial even if it is statistically significant. An effect size of 0.8 is considered large [30].

## Results

Cronbach’s coefficient Alpha for the MDAS in the current study was 0.89 indicating that the data on the MDAS for the student dental sample is reliable. The lowest and highest scores possible on the MDAS questionnaire are 5 and 25 respectively. The mean score from all respondents was 10.76 (*SD =* 5.06). For comparative purposes and to assess hypothesis 1, Table 1 presents normative data for the MDAS adapted from Humphris, Freeman, Campbell, Tuutti and D’Souza [25].

**Table 1:** Comparison of mean Student Dental Clinic MDAS scores with normative samples from different geographical locations

	Belfast (200)		Dubai (200)		Helsinki (200)		Jvaskyla (194)		All samples to the left (794)		Student Dental Clinic (102)	
	Mean	SD	Mean	SD	Mean	SD	Mean	SD	Mean	SD	Mean	SD
Visiting Tomorrow	2.43	1.42	1.66	0.82	1.72	0.91	1.81	1.00	1.91	1.11	1.88	1.14
Waiting Room	2.44	1.35	1.81	1.01	1.80	0.93	1.94	1.02	1.99	1.12	1.92	1.15
Tooth Drilled	2.92	1.48	2.29	1.28	2.25	1.12	2.51	1.23	2.49	1.31	2.61	1.23
Scale and Polish	1.90	1.35	2.27	1.08	1.87	1.04	1.84	0.99	1.96	1.09	1.89	1.18
LA Injection	2.76	1.45	2.83	1.18	1.81	0.65	2.44	1.21	2.45	1.23	2.46	1.34
MDAS	12.40	5.98	10.90	4.28	9.44	3.91	10.54	4.65	11.27	5.07	10.76	5.06
Percentage scoring 19 or above	19.50%		6.00%		3.00%		8.80%		9.30%		9.90%	
Cronbach’s Alpha	0.90		0.86		0.89		0.88		0.89		0.89	

Adapted from: Humphris et al. [25]

### Patient differences on the MDAS

In regard to hypothesis 2, the mean MDAS for males was 9.94 (*SD* = 4.99), and for females 11.93 (*SD* = 5.14). The statistical significance of the mean difference between the genders on total MDAS scores at pre-dental treatment was enumerated to be t_(97,89)_ = − 1.93, *p =* 0.056. For each of the MDAS items, females had a slightly higher mean anxiety than the male respondents (Table 2). Of the MDAS items, Local Anaesthetic injection and Having a Tooth Drilled, produced the highest mean scores for provoking anxiety. There was a significant difference between males and females for the item ‘tooth drilled’ *t*_(97,84)_ = − 2.14, *p* = .03. Females had more anxiety in regard to having a tooth drilled than did males (*M* = 2.93, *SD* = 1.29 compared to *M* = 2.39, *SD* = 1.15).

**Table 2:** Gender differences on the MDAS items (*N* = 99)

	Gender	N	Mean	Std. Deviation	Std. Error Mean
Treatment tomorrow	Male	56	1.71	1.004	.134
	Female	43	2.12	1.295	.197
Waiting Room	Male	56	1.80	1.102	.147
	Female	43	2.09	1.231	.188
Tooth Drilled	Male	56	2.39	1.155	.154
	Female	43	2.93	1.298	.198
Scale and Polish	Male	56	1.79	1.074	.144
	Female	43	2.02	1.336	.204
LA Injection	Male	56	2.25	1.283	.171
	Female	43	2.77	1.411	.215

To test hypothesis 3 a oneway ANOVA was used to determine if there were significant age differences on the MDAS scores at pre-treatment. The age groupings were age less than and including 30 years, 31–50 years and 51 plus years. A significant difference for age was found on the dependent variable of MDAS score at pre-treatment *F*_(2,96)_ = 3.24, *p* < .05. Table 3 presents the mean MDAS scores for each age group. Patients aged less than 30 years had the highest dental anxiety scores (*M* = 12.15, *SD* = 5.47) while those aged over 50 years had the lowest dental anxiety (*M* = 9.34, *SD* = 4.57). The item which distinguished the age groups on dental anxiety was anticipating having a tooth drilled *t*_(2,96)_ = 3.97, *p* = .02. Younger adults had more anxiety (*M* = 3.10, *SD* = 1.16) compared to those aged over 50 years (*M* = 2.26, *SD* = 1.09).

**Table 3:** Mean differences for age on MDAS scores at pre-treatment (*N* = 81)

Age	N	Mean	s.d.	Lower bound 95% confidence interval	Upper bound 95% confidence interval
Under 30 years	20	12.15	5.47	9.58	14.71
31–50 years	36	11.80	5.26	10.02	13.58
51 plus years	43	9.34	4.57	7.94	10.75

### Pre-post treatment differences on anxiety

Participants completed a post-treatment survey in order to make a comparison between pre-treatment self assessed waiting room anxiety levels, and post-treatment self assessed anxiety level. Data was explored for factors which attenuated anxiety levels and those which compounded anxiety levels, including dental clinic environment factors and treatment undergone.

A single question on the anxiety of participants was answered before (MDAS Waiting Room question 2: Sitting in the waiting room how anxious do you feel?) and after treatment (How anxious are you feeling at the moment?) with a numerical value corresponding to the respondents’ experience of anxiety; 1 = not anxious, 2 = slightly anxious 3 = fairly anxious 4 = very anxious 5 = extremely anxious. Hypothesis 4 was analysed using a repeated measures ANOVA. The mean score from the pre-treatment question was 1.92 (*SD* = 1.15), in comparison to the post-treatment mean score of 1.23 (*SD* = 0.64). There was a significant effect for time (pre/post treatment), calculated using Wilks’ Lambda = .25, *F* (1,100) = 39.28.17, *p* < .0005, multivariate partial eta squared = .282. The partial eta squared result of .282 is indicative of a large effect of time, using Cohen’s guidelines [30]. The self reported anxiety levels of respondents were significantly less post-treatment.

The post-appointment questionnaire asked participants which treatment they had received in order to evaluate hypothesis 5 that complex treatments would evoke higher levels of anxiety. Complex treatments were primarily defined as those that required a local anaesthesia injection.

Table 4 shows that the majority of patients visited the clinic for a first check-up and intra-oral x-rays, scale and clean, and diagnostic pulp testing. Most of the treatments received were not invasive. There was no significant difference on post-appointment anxiety levels between those patients receiving invasive complex treatments versus those who had received non-invasive treatment t(99,96) = −.263, n.s. However, those waiting to receive a complex treatment had higher MDAS scores (M = 11.96, *SD* = 5.49) than those waiting to receive a non-invasive treatment (M = 9.18, *SD*. = 3.94) t(100,99) = − 2.97, *p* = .004.

**Table 4:** Frequency and Percentage of patients receiving each type of treatment at the first appointment (*N* = 102)

Treatment Received	Frequency	Percentage (%)
First check up	83	81.4
Limited first appointment, checking for specific problem	11	10.8
Removal of Pain	11	10.8
One or more X-rays	54	52.9
Pulp testing	24	23.5
Scale and Clean	38	37.3
Scale and Clean under gums, requiring LA	4	3.9
Varnish to reduce tooth sensitivity	4	3.9
Fluoride Varnish	5	4.9
Denture Appointment	4	3.9
Treating Gum disease	1	1
Extraction of tooth	6	5.9
Beginning RCT	3	2.9
Tooth coloured filling	10	9.8
Metal Filling	3	2.9

Included in the post-treatment survey were two questions to enumerate the factors which made the respondents more anxious and those which made the patient less anxious. These factors were Time length of appointment, Clinical environment, perceived Interpersonal skill of the student, perceived Clinical ability of the student, Presence of the supervisor, Having to participate in the procedure (holding suction), Knowledge of additional appointments. It was found that 71.6% of the patients did not find any of the factors mentioned as making them more anxious. As can be seen form Table 5, the perceived interpersonal skills of the student had the most influence in reducing anxiety in patients, with 50% of patients citing this factor as making them less anxious.

**Table 5:** Percentage of patients indicating different levels of anxiety to different dental surgery factors post-treatment (*N* = 102)

	More Anxious	Less Anxious
Time Length of Appointment	12 (11.8%)	14 (13.7%)
Clinical Environment	4 (3.9%)	29 (28.4%)
Interpersonal Skills of the Student	3 (2.9%)	51 (50%)
Clinical ability of the student	5 (4.9%)	41 (40.2%)
Presence of the Supervisor	3 (2.9%)	31 (30.4%)
Having to participate in the procedure (holding suction)	4 (3.9%)	5 (4.9%)
Knowledge of Additional appointments	9 (8.8%)	25 (24.5%)
None of the Above	73 (71.6%)	32 (31.4%)
Other	8 (7.8%)	5 (4.9%)

Post survey results showed that in addition to the perceived ‘Interpersonal Skills of the Student’, the dental surgery factor perceived ‘Clinical ability of the student’ was cited by 40.2% of patients as reducing anxiety levels. Having the dental Supervisor present to check and advise on procedures (30.4%) and the clinical environment of the dental surgery (28.4%) were also rated by patients as lessening anxiety. The time length of the dental appointment was cited most often as making patients more anxious. The longer the dental procedure the more anxious the patient.

## Discussion

The aim of the study was to document anxiety levels from new patients at a Student Dental Clinic using the MDAS, explore pre-post treatment differences in dental anxiety and the factors which may contribute to, or reduce anxiety. With minor exceptions [10, 21–24] most of the existing literature on dental anxiety has been conducted on dental practitioners rather than on student dentists. This study therefore afforded comparisons between the dental anxiety experiences of patients undergoing treatment from a student dentist compared to a registered experienced dentist.

The first hypothesis that patients at the Student Dental clinic would experience higher levels of anxiety on the MDAS compared to normative samples [25] was not supported. When the results from Student Dental Clinic patients (no gender differentiation) were compared with those documented by Humphris et al. [25] (Table 1) from varying geographical locations, the results rendered similar trends with no significant irregularities. The current reported anxiety levels using the MDAS were relatively similar to normative data from the different regions. The patients in our study had anxiety levels comparable to those of patients attending admission clinics in dental hospitals from four cites in three different countries, undergoing procedures by experienced dental practitioners [25].

There was some support for hypothesis 2 of gender differences on dental anxiety. Previous research [2, 3] in Australia has reported females to have more fear of the dentist than males. This is in accordance with our results showing females had a higher mean MDAS score. An explanation for this may be that females report lower pain thresholds and tolerance toward pain [31]. Similar findings have been reported elsewhere in anxiety and fear studies. Holtzman and colleagues [18] found that women had more fear of specific stimuli such as injection or tooth drilling. In our study females similarly reported higher levels of anxiety for the specific treatments of ‘LA injection’ and ‘tooth drilling’. Treatment involving injections and vibrating sensations are generally associated with pain especially in the dentally anxious [14, 15].

Several studies have suggested that there is an inverse relationship between age and levels of dental anxiety [6, 9]. This has not been shown to be a universal finding [3]. However in the present study dental anxiety was found to be lower in the older age groups, thus supporting hypothesis 3. Younger age groups reported higher means for MDAS consistent with the research [6] which has found an increase in anxiety in the early adult years. This may be due to increased exposures over time allowing patients to develop a tolerance to treatment, and therefore have less anxiety as they age.

There was a significant difference in dental anxiety from pre to post-treatment on the single item anxiety measure thus supporting hypothesis 4. The lower mean anxiety level from the post dental treatment surveys is suggestive of a positive experience at the Student Dental Clinic. Patients’ anticipatory experience of anxiety was higher than the anxiety experience after having undergone treatment at the student dental clinic. Previous research [10, 21–24] on patients attending a student dental clinic has reported high anxiety levels. One study [10] on the experience of patients in a student clinic reported 58% as having dental anxiety, whereas our sample reported relatively low dental anxiety (9.9%). However, it is not known what types of treatment were received by the patients in the Isfahan Dental School.

In the current study the complexity of treatment was not found to be associated with higher levels of anxiety. Hypothesis 5 was therefore not supported. The literature indicates that invasive procedures such as subgingival scaling, deep probing, extractions and root canal therapy are associated with higher reported anxiety [8, 14]. However, the majority of patients in the current study underwent a check-up appointment with one or more x-rays taken, so it seems unlikely that this would increase patient anxiety toward dental treatment.

The relatively low levels of anxiety post-treatment could be due to the lack of invasiveness of procedures, or the patients’ expectations and fear being met with a relatively pleasant treatment. Or perhaps these new patients were quite concerned about the possibility that the dental examination/xrays would reveal many and/or large dental problems, and responded with relief to the news that things were not as bad as they may have feared. It has been suggested that "worry about what the dentist will find" makes some anxious individuals put off going to the dentist. Another possibility is that some of the new patients who were dentally-anxious experienced relief that the dental encounter was nearly over, as their appointments were completed and they were leaving the dental environment. An additional possibility is that extremely anxious patients did not participate in the study. However, this potential source of selection bias does not seem likely as all participants who were approached participated in the study.

The post survey results showed that the perceived ‘Interpersonal Skills of the Student’ and perceived ‘Clinical ability of the student’ were most often indicated as making the patients less anxious. This suggests that the patients perceive the students to have an adequate level of skill to perform treatment, and that they are able to conduct themselves in a welcoming manner. Recent research [19] has found that negative evaluations of previous dental experiences influence the behavioural intention to visit the dentist as a result of expectations of an unpleasant or painful future experience. The current findings indicate that the perceived interpersonal skills of the student dentist in making the patient feel at ease, and in conveying confidence in the delivery of the treatment, may serve to lessen negative evaluations of the dental experience. Patients may also have felt less anxiety given that a Clinical Supervisor was present during the procedure. However, student dentists are under the supervision of a qualified Clinical Supervisor at a ratio of 8:1, which can prolong treatment times which may differ from patients’ previous experiences with dental treatment. The time length of the appointment was the most frequently indicated item for making the patient more anxious.

### Limitations

The complexity of treatments received by the participants is a limitation to this study, as third year undergraduate students, who are unable to practise the full scope of dental procedures, primarily treated the participants. The treatments these 3rd year undergraduate students are able to perform are limited and do not include removal of teeth, posterior endodontics, or crown and bridge work. The procedures, which are reserved for fourth and fifth year students, in addition to qualified dental practitioners, are in most cases more complex. This study defined complex treatments as those which required local anaesthesia, excluding treatments such as ‘impression taking’, which could potentially be just as anxiety provoking for the patient. Furthermore, all participants were new patients to the Student Dental Clinic. If existing patients were included, it is likely that a greater range and hence greater complexity of treatment procedures would have been noted.

## Conclusions

This study confirmed gender and age differences in dental anxiety. There was a reduction in dental anxiety from pre-treatment to post-treatment though most of the treatment being undergone by patients was for less complex procedures. Of the dental surgery environment factors, this study found that effective interpersonal skills and the perceived clinical ability of the student dentist were facilitative of reduced anxiety in patients. It is recommended that Clinical Supervisor-student ratios need to be more equivalent in order to reduce the time length of appointments which currently is associated with increased patient anxiety levels in dental clinics.

## Abbreviation

MDAS: Modified Dental Anxiety Scale
